# Exploring the potential of ChatGPT as an adjunct for generating diagnosis based on chief complaint and cone beam CT radiologic findings

**DOI:** 10.1186/s12911-024-02445-y

**Published:** 2024-02-19

**Authors:** Yanni Hu, Ziyang Hu, Wenjing Liu, Antian Gao, Shanhui Wen, Shu Liu, Zitong Lin

**Affiliations:** 1 Department of Dentomaxillofacial Radiology, Nanjing Stomatological Hospital, Affiliated Hospital of Medical School, Institute of Stomatology, Nanjing University, Nanjing, Jiangsu, People’s Republic of China; 2 Department of Stomatology, Shenzhen Longhua District Central Hospital, Shenzhen, People’s Republic of China

**Keywords:** Large language model, CBCT, Dental Disease, Neoplastic/cystic diseases, Radiologic finding, Radiologic impression, Diagnosis

## Abstract

**Aim:**

This study aimed to assess the performance of OpenAI’s ChatGPT in generating diagnosis based on chief complaint and cone beam computed tomography (CBCT) radiologic findings.

**Materials and methods:**

102 CBCT reports (48 with dental diseases (DD) and 54 with neoplastic/cystic diseases (N/CD)) were collected. ChatGPT was provided with chief complaint and CBCT radiologic findings. Diagnostic outputs from ChatGPT were scored based on five-point Likert scale. For diagnosis accuracy, the scoring was based on the accuracy of chief complaint related diagnosis and chief complaint unrelated diagnoses (1–5 points); for diagnosis completeness, the scoring was based on how many accurate diagnoses included in ChatGPT’s output for one case (1–5 points); for text quality, the scoring was based on how many text errors included in ChatGPT’s output for one case (1–5 points). For 54 N/CD cases, the consistence of the diagnosis generated by ChatGPT with pathological diagnosis was also calculated. The constitution of text errors in ChatGPT’s outputs was evaluated.

**Results:**

After subjective ratings by expert reviewers on a five-point Likert scale, the final score of diagnosis accuracy, diagnosis completeness and text quality of ChatGPT was 3.7, 4.5 and 4.6 for the 102 cases. For diagnostic accuracy, it performed significantly better on N/CD (3.8/5) compared to DD (3.6/5). For 54 N/CD cases, 21(38.9%) cases have first diagnosis completely consistent with pathological diagnosis. No text errors were observed in 88.7% of all the 390 text items.

**Conclusion:**

ChatGPT showed potential in generating radiographic diagnosis based on chief complaint and radiologic findings. However, the performance of ChatGPT varied with task complexity, necessitating professional oversight due to a certain error rate.

## Introduction

The advent of artificial intelligence (AI) has brought about significant changes in various fields, most notably in medical field [1–5]. Of these AI models, AI-driven large language models (LLMs), trained on vast text corpora, have the capability to effortlessly produce high-quality text (and software) across a diverse range of subjects [6, 7].

The Generative Pre-trained Transformer (GPT), a subclass of large language models, was developed by OpenAI [8]. This versatile model can be adapted to various linguistic tasks, ranging from language translation and text summarization to text completion [9]. As one of its latest versions, ChatGPT as GPT version 3.5, has an impressive 175 billion parameters, making it more powerful than its predecessors [10]. ChatGPT has the potential to revolutionize the medical field specifically radiology, thereby reducing the workload of radiologists [11].

Recently, several studies explore the potential usage of ChatGPT in radiology reports writing or translation [12–16] (Table 1). Mago and Sharma [13] evaluated the potential usefulness of ChatGPT-3 in oral and maxillofacial radiology for report writing by identifying radiographic anatomical landmarks, and learning about oral and maxillofacial pathologies, and their radiographic features. Doshi et al. [14] and Jeblick et al. [15] used ChatGPT to simplify radiology reports to enhance the readability of radiology reports. Lyu et al. [16] also explored the feasibility of using ChatGPT to translate radiology reports into plain language for patients and healthcare providers. It showed that potential usage of ChatGPT in radiology reports. However, there is no research exploring whether ChatGPT can generate diagnostic conclusions based on patient’s chief complaint and imaging findings.

**Table 1:** Researches of large language models (LLMs) in radiology in 2023

No	Author(Year)	Methods	Sample size	Type of LLMs	Prompt	Scoring method
1	Mago and Sharma (2023)	Asking ChatGPT about radiographic anatomical landmarks, oral and maxillofacial pathologies, and their radiographic features	A questionnaire consisting of 80 questions	ChatGPT3	No prompt	4-point modified Likert scale
2	Doshi et al. (2023)	Simplify radiology reports	254 radiology reports	ChatGPT3.5, ChatGPT4.0, Google Bard, and Microsoft Bing	Three types of prompts	Readability scores
3	Jeblick et al. (2023)	Simplify radiology reports	3 radiology reports	ChatGPT	One prompt	5-point Likert scale
4	Lyu et al. (2023)	Translate radiology reports into plain language	62 chest CT and 76 brain MRI reports	ChatGPT4	Seven types of prompts	5-point Likert scale

It’s well-established that normal radiology reports comprise two main sections, radiologic findings and radiologic impression. The radiologic findings provide objective and detailed image descriptions of the lesions. For neoplastic or cystic diseases, it often includes information about the location, extent, size, density, boundary, and shape of lesions. The radiologic impression offers diagnostic conclusions based on the chief complaint and the radiologic findings. According to the chief complaint and radiologic findings, the radiologist draws the final diagnosis conclusion, which is also called radiologic impression. The impression usually includes the diagnosis related to the chief complaint (based on both the patient’s chief complaint and the radiologic findings), as well as the diagnosis unrelated to the chief complaint (based solely on the radiologic findings). Moreover, it often includes a range of differential diagnoses. In clinical practice, providing a radiologic diagnosis is based on the chief complaint and radiologic findings. However, numerous diseases may exhibit similar chief complaints and radiologic findings and the same diseases may have different radiologic findings. Thus, diagnosing based on these requires a high degree of clinical acumen and years of specialized training [17]. For many young radiologists, delivering an accurate, complete, and logical diagnosis can be a challenging task. As of yet, there is insufficient evidence to substantiate ChatGPT’s capability in generating diagnosis based on chief compliant and radiologic findings [18].

Therefore, in this study, we aim to investigate the utility of ChatGPT in generating diagnostic conclusions based on patient’s chief complaints and CBCT radiologic findings. Specifically, the diagnosis accuracy, diagnosis completeness, and text quality of ChatGPT’s performance were evaluated. We believe that this research will provide valuable insights into the potential and limitations of using AI language models in generating diagnosis (image impressions) for radiologic reports.

## Materials and methods

**Fig. 1:**
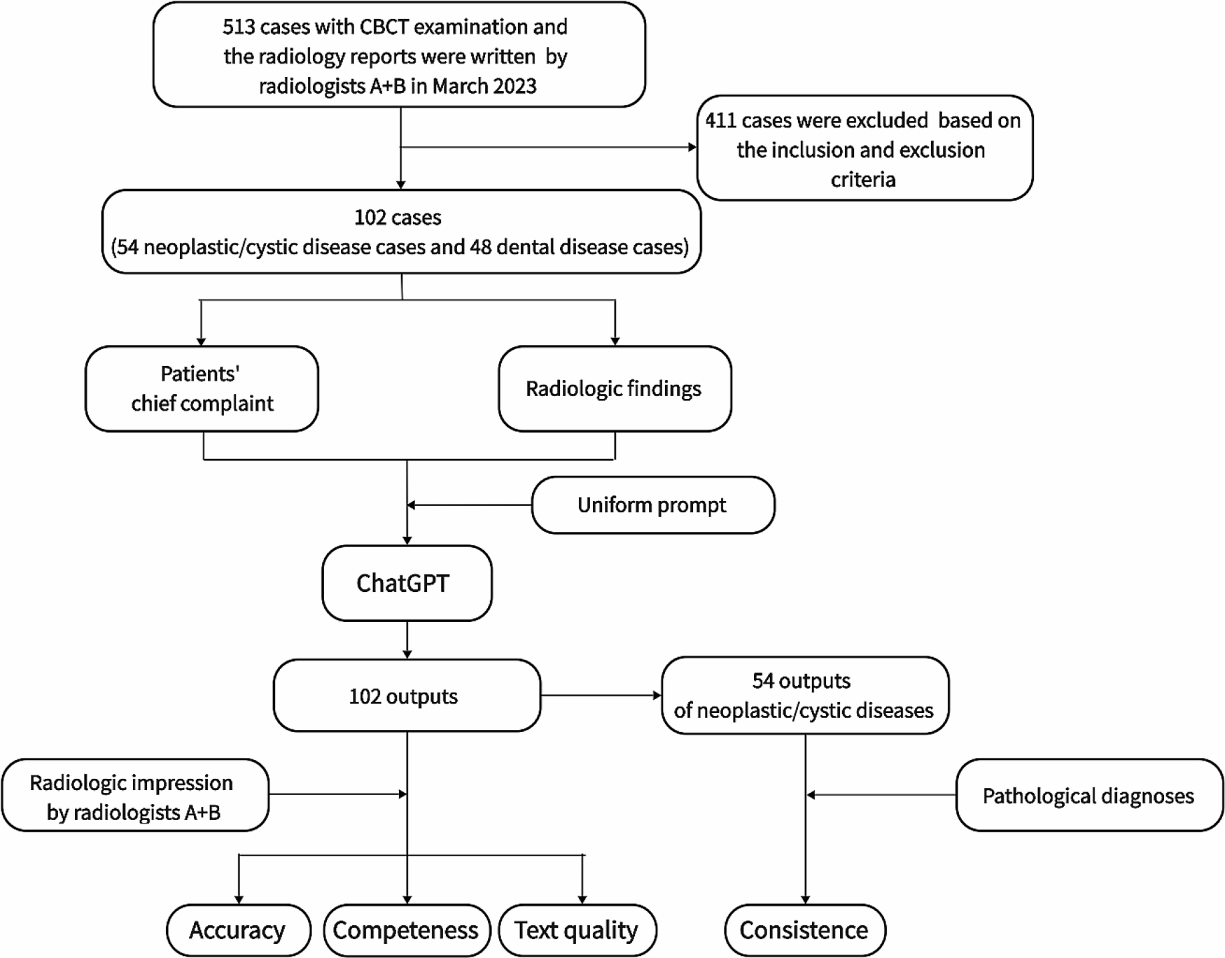
The flow chart of the study protocol

A schematic workflow of this study was showed in Fig. 1.

### Patients and datasets

The whole CBCT volume and reports were retrieved from the picture archiving and communication system (PACS) of our hospital. The inclusion criteria were as follows: (1) patients whose chief complaints are clearly documented in the Electronic Medical Record (EMR) system; (2) the CBCT image presented either dental diseases (DD) or neoplastic/cystic diseases (N/CD); (3) final diagnoses included a chief complaint related diagnosis and one diagnosis or more diagnoses unrelated to the chief complaint (based on the radiologic findings); (4) for N/CD, definite pathological diagnosis was available after surgery. The exclusion criteria were as follows: (1) the CBCT images taken for orthodontic or implant purposes; (2) CBCT images of poor quality, exhibiting motion artifacts or foreign body artifacts; (3) radiology reports containing only a single diagnostic impression. To ensure reliability and consistency of CBCT reports, all the CBCT reports including radiologic findings and radiologic impressions were written by one radiologist with 10 years of experiences (Radiologist A) and reviewed and modified by another radiologist with 15 years of experiences (Radiologist B) to ensure dental-specific terminology were used. In total, 102 CBCT reports were retrospectively collected, comprising 48 focused on DD and 54 on N/CD.

All patients’ protected health information (name, gender, address, ID number, date of birth, personal health number) was verified to be excluded from the input of ChatGPT. The approval from the Ethics Committee of the Nanjing Stomatological Hospital, Medical School of Nanjing University was obtained prior to perform this study.

### Optimization of ChatGPT input prompts

**Fig. 2:**
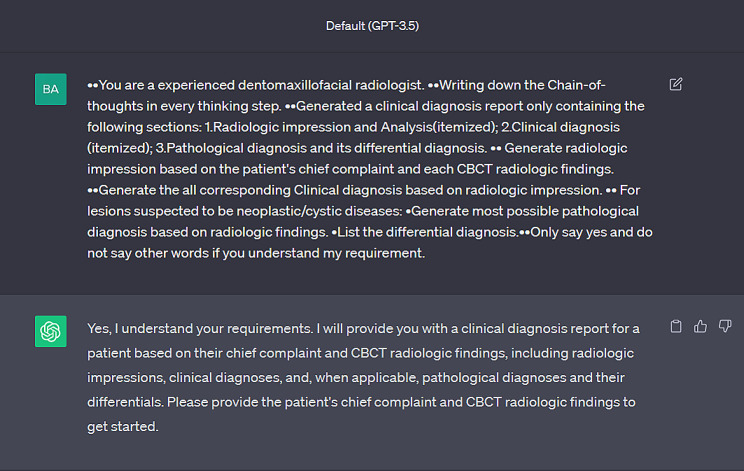
Prompt engineering of ChatGPT’s input

The prompt engineering is crucial for optimizing the performance of the LLM [19]. To enhance the accuracy and completeness of diagnostic outputs of ChatGPT, the following strategies were used:

1. The prompt was used for all the cases (Fig. 2). The prompt was as following:

- You are an experienced dentomaxillofacial radiologist.
- Writing down the Chain-of-thoughts in every thinking step.
- Generated a clinical diagnosis report only containing the following sections: 1.Radiologic impression and Analysis(itemized); 2. Clinical diagnosis (itemized); 3. Pathological diagnosis and its differential diagnoses.
- Generate radiologic impression based on the patient’s chief complaint and each CBCT radiologic findings.
- Generate all corresponding clinical diagnoses based on radiologic impression.
- For lesions suspected to be neoplastic/cystic diseases: 1.Generate most possible pathological diagnosis based on radiologic findings. 2. List the differential diagnoses.
- Only say yes and do not say other words if you understand my requirement.

2) The prompts were initially input into ChatGPT. After receiving a response, the chief complaint and the CBCT radiologic findings were inputted together. These inputs were performed by a radiologist with 3 years of experiences (Radiologist C).
3) For each individual case, reset the chat interface to eliminate the influence of preceding interactions on the model’s output.

Initially, 10 cases (comprising 5 N/CD cases and 5 DD cases) were utilized to optimize ChatGPT’s input prompts. Various prompts were tested until the output diagnostic conclusions for these 10 cases demonstrated relatively high completeness, stability, and accuracy. The prompt that met these criteria was then selected as the final one.

### Evaluation of ChatGPT’s outputs

The diagnoses generated by ChatGPT were assessed utilizing the five-point Likert scale. For diagnosis accuracy, the scoring was based on the accuracy of chief complaint related diagnosis and chief complaint unrelated diagnoses; for diagnosis completeness, the scoring was based on how many accurate diagnoses included in ChatGPT’s output for one case; for text quality, the scoring was based on how many text errors included in ChatGPT’s output for one case. The radiologic impressions formulated by Radiologist A, and subsequently reviewed and modified by Radiologist B, were used as the benchmark results. The diagnosis scoring was conducted by two radiologists (Radiologist C and Radiologist D). The scoring of Radiologist C was used as the evaluation scores of this study. The inter-rater reliability of ChatGPT’s output evaluation was calculated between Radiologist C and Radiologist D. Before evaluation, standardized training of the five-point Likert scaling was performed for Radiologist C and Radiologist D (Detailed definition showed in Table 2).

**Table 2:** The scoring for ChatGPT’ s diagnosis output

Score	Accuracy	Completeness	Text quality
**1**	All diagnosis is incorrect	0-20% diagnoses are included	More than 5 text errors
**2**	Chief complaint related diagnosis is incorrect; Partial chief complaint unrelated diagnoses are correct	20-40% diagnoses are included	3 ~ 4 text errors
**3**	Chief complaint related diagnosis is incorrect; All chief complaint unrelated diagnoses are correct	40-60% diagnoses are included	2 text errors
**4**	Chief complaint related diagnosis is correct; Partial chief complaint unrelated diagnoses are correct	60-80% diagnoses are included	1 text error
**5**	All diagnoses are correct	80-100% diagnoses are included	No text error

For the 54 N/CD cases, retrospective collection of postoperative pathological diagnoses was also performed. A radiologist with 1 years of experience (Radiologist E) reviewed the chief complaint and the CBCT radiologic findings and gave diagnoses for these 54 N/CD cases. The diagnosis of ChatGPT’s, Radiologist A + B, and Radiologist E compared with the final pathological diagnosis.

## Results

### ChatGPT’s diagnosis accuracy, diagnosis completeness and text quality

**Fig. 3:**
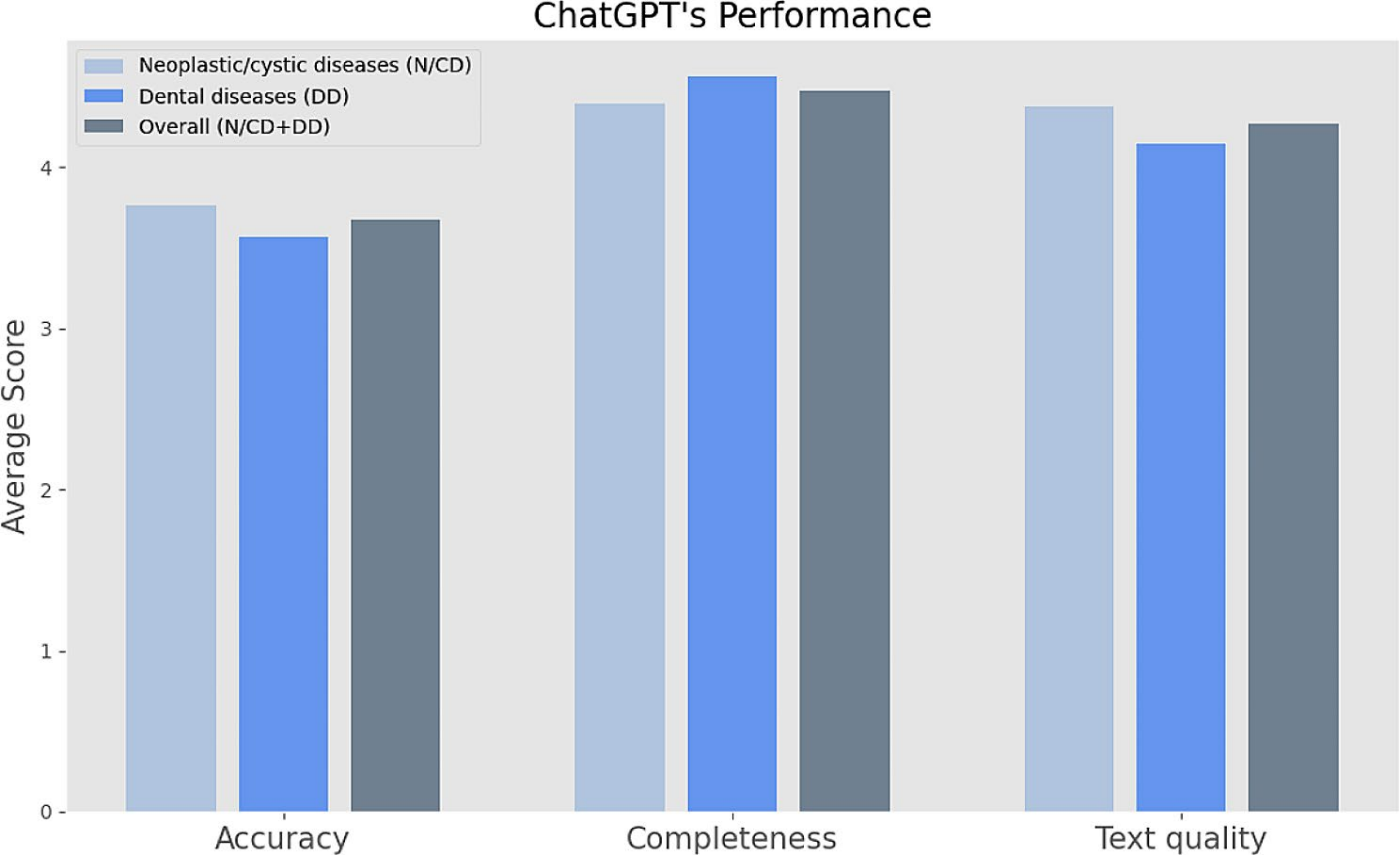
Histogram of ChatGPT in terms of accuracy, completeness and text quality

For all the 102 diagnostic outputs generated by ChatGPT, the accuracy, completeness and text quality scores were 3.7 (out of 5), 4.5 (out of 5), and 4.6(out of 5) respectively (Table 3; Fig. 3).

**Table 3:** The score distribution for ChatGPT’ s performance

	Accuracy		Completeness		Text Quality	
	N/CD	DD	N/CD	DD	N/CD	DD
Score 5	20	11	33	36	43	28
Score 4	13	17	11	7	5	17
Score 3	13	10	8	4	5	3
Score 2	4	8	2	1	1	0
Score 1	4	2	0	0	0	0
Mean	3.8	3.6	4.4	4.6	4.7	4.5
Mean (102 cases)	3.7		4.5		4.6	
Inter- examiner agreement	0.890		0.801		0.778	

N/CD: Neoplastic/cystic diseases; DD: Dental diseases

### Comparison of ChatGPT and radiologists’ diagnoses with pathological diagnosis for N/CD

**Fig. 4:**
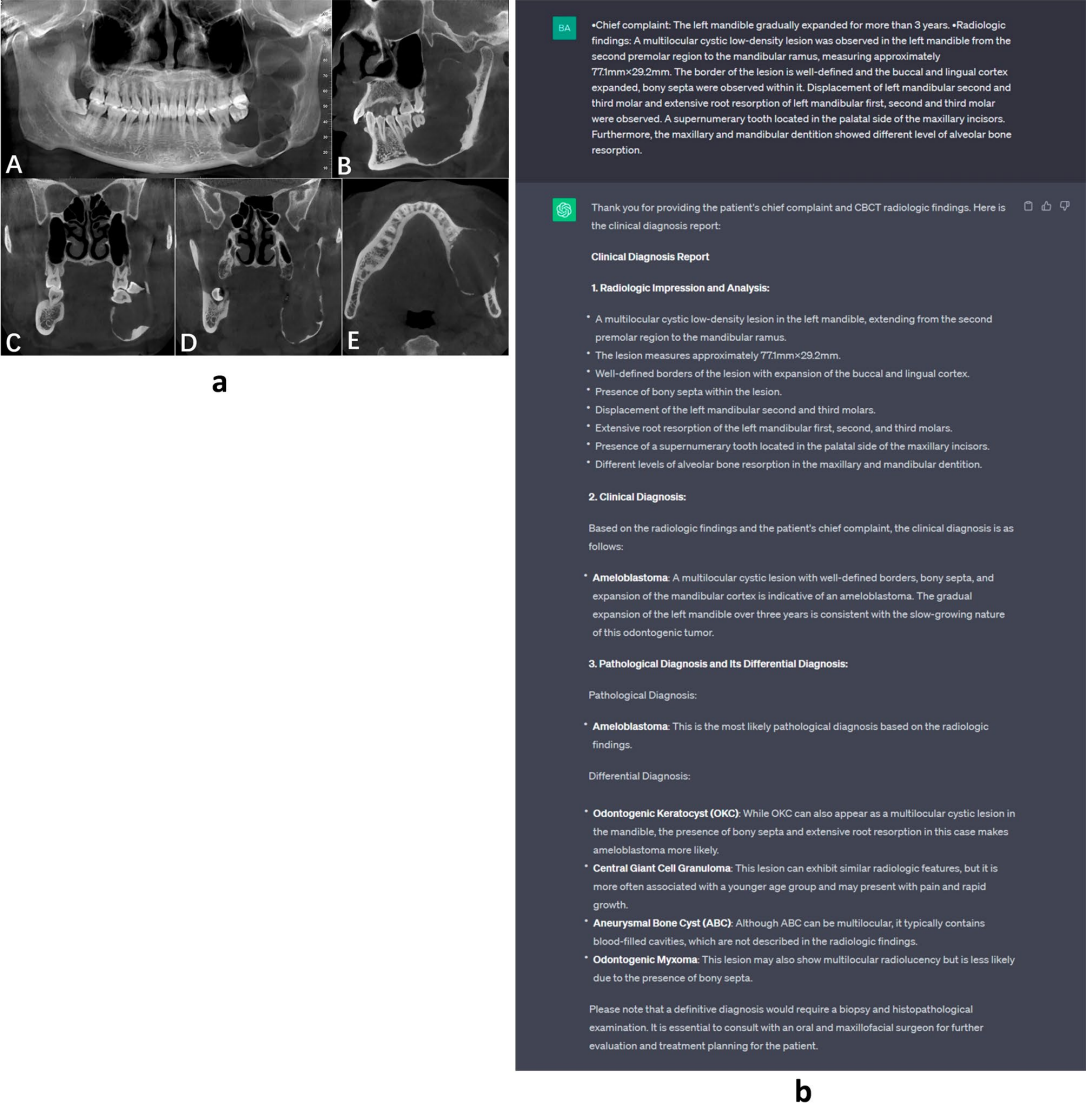
**A** The CBCT presentation of a mandibular ameloblastoma; **B** The input and output in ChatGPT

The pathological classifications for the 54 N/CD cases were displayed in Table 4. Within this subset, ChatGPT all offered multiple potential diagnoses. Of them, ChatGPT’s first diagnosis (the most likely pathological diagnosis) aligned with pathological diagnosis in 38.9% of the cases (Fig. 4A and B); one of ChatGPT’s diagnoses (not the first diagnosis) coincided with the pathological diagnosis in 31.5% of the cases; conversely, ChatGPT’s diagnoses were inconsistent with pathological diagnosis at all in 29.6% of the cases. For radiologist A + B, the first diagnosis aligned with pathological diagnosis in 48.1% cases; for radiologist E, the first diagnosis aligned with pathological diagnosis in 31.5% of cases (Table 5).

**Table 4:** The pathological types of the 54 neoplastic/cystic diseases

Type of diseases	NO.
Odontogenic cyst	24
Odontogenic keratocyst	5
Ossifying fibroma	7
Ameloblastoma	5
Squamous cell carcinoma	4
Osteoma	2
Osteomyelitis of jaw	2
Odontoma	2
Nasopalatine cyst	1
Osteosarcoma	1
Calcifying epithelial odontogenic tumor	1
Total	54

**Table 5:** Comparison of ChatGPT, radiologist A + B and radiologist E’s diagnosis with the final pathological diagnosis

	No. (Percentage)		
	ChatGPT	Radiologist A + B	Radiologist D
The first diagnosis is consistent with the pathological diagnosis	21(38.9%)	26(48.1%)	17(31.5%)
One of diagnoses matches pathological diagnosis	17(31.5%)	20(37.0%)	18(33.3%)
None of the diagnosis is consistent with the pathological diagnosis	16(29.6%)	8(14.8%)	19(35.2%)

Note: There are totally 54 neoplastic/cystic diseases

### ChatGPT’s text errors

Given ChatGPT’s outputs are long and serial numbers are presented, we segmented the text data of each case into 3–4 text items based on the serial numbers. This process resulted in a total of 390 text items. Of these, 88.7% (346 out of 390) were error-free. However, errors were found in 44 text items. Among these errors, 63.6% involved imprecise dental terminology, 29.5% were hallucinations, and 6.8% included imprecise descriptions of disease location (Table 6).

**Table 6:** Text error for ChatGPT’s diagnosis

		Number of answers (percentage)	Number of mistakes (percentage)
Without text error		346 (88.7%)	0
Text error	Imprecise language	44 (11.3%)	28 (63.6%)
	Hallucination		13 (29.5%)
	Wrong location		3 (6.8%)

Note: There are totally 390 text items

### Discussion

The integration of AI into the field of radiology has been a topic of considerable interest in recent years [20, 21]. The potential of AI to revolutionize the generation of radiology reports is immense. However, the current version of ChatGPT serves as a text-based AI model and thus not able to analyze radiologic images directly. And direct radiology reports generation using ChatGPT is currently impractical. But if we input the chief complaint and detailed radiologic findings, ChatGPT could generate potential diagnoses. Therefore, in this research, we utilized OpenAI’s ChatGPT, a state-of-the-art language model, to generate diagnoses for CBCT images based on chief complaints and CBCT radiologic findings, evaluating its diagnosis accuracy, completeness, and text quality in the process. Our results revealed a promising yet complex picture of the capabilities and limitations of ChatGPT in this context.

In our study, the scores obtained by ChatGPT were 3.6/5 for accuracy, 4.5/5 for completeness, and 4.6/5 for text quality. These scores provide a quantitative measure of ChatGPT’s performance. Completeness emerged as a strong point in ChatGPT’s performance. An impressive 97.1% (99 out of 102) of the reports scored 3 or higher on the completeness scale and 67.6% (69 out of 102) scored the maximum points. This suggests that ChatGPT was able to provide comprehensive diagnostic opinion based on the provided information. The completeness score for DD, with a composite score of 4.6, was slightly higher than for N/CD, which had a composite score of 4.4. This could indicate the model’s ability to handle the complexity of DD, despite the challenges posed by the need for precise CBCT image descriptions. In terms of text quality, 99.0% (101 out of 102) of the reports scored 3 or higher. The text quality score for DD (4.5) was slightly lower than for N/CD (4.7). This could be indicative of the challenges posed by the specific dental terminology required for dental diseases.

In terms of diagnostic accuracy, ChatGPT achieved a score of 3.7 across all 102 cases. These cases, which were all relatively complex instances in the oral and maxillofacial region, were specifically selected for this study. Cases related to orthodontics or implants, as well as those with only a single diagnostic impression, were excluded. Consequently, a final diagnostic accuracy score of 3.7 points was attained. Moreover, ChatGPT performed slightly better for N/CD, with a composite score of 3.8, compared to DD, which had a composite score of 3.6. This variation in performance could be attributed to the nature of radiologic impression provided for different diseases. Radiologists often provide a more general diagnosis for N/CD, which might have eased ChatGPT’s task of generating diagnosis. On the other hand, dental diseases often require more precise diagnoses, which might have posed a greater challenge for the ChatGPT. This suggests that the performance of ChatGPT will vary depending on the complexity and specificity of the task.

Regarding the capability for ChatGPT to directly generate a pathological diagnosis for neoplastic/cystic diseases, the model’s performance was found to be less satisfactory. Of the 54 cases, the first diagnosis aligned with pathological diagnosis in 38.9% of the cases for ChatGPT’s, in 48.1% cases for radiologist A + B and in 31.5% of cases for radiologist E. The performance of ChatGPT was inferior to that of experienced radiologists A and B, yet it outperformed radiologist E, who has fewer years of experience. This suggests that formulating a pathological diagnosis remains challenging, especially for radiologists with fewer years of experience in clinical practice. ChatGPT may struggle with complex medical problems, and highlights the need for caution when using AI models for complex diagnostic purposes.

In our study, 88.7% text items were error-free. However, there still existed an 11.3% error rate in text items, encompassing imprecise dental terminology (63.6%), hallucinations (29.5%), and imprecise description of disease location (6.8%). For imprecise language and misinterpretation of medical terms, these could be attributed to the model’s limited exposure to dentistry-related training data, resulting in gaps in its understanding of this specialized field. Hallucinations are a common issue among natural language generation models. The term “hallucination” refers to a phenomenon where the model generates text that is incorrect, nonsensical, or unreal. It’s a widespread challenge encountered by many natural language processing models [22, 23]. Furthermore, ChatGPT tends to follow instructions rather than engage in genuine interaction [24]. For instance, when the radiologic findings are insufficient, ChatGPT may make assumptions that cannot be derived from the radiologists’ descriptions. While ChatGPT has shown impressive capabilities in generating human-like text, its application in specialized fields like radiology may require additional oversight. Given the complexity of radiology and possible errors in AI-generated diagnostic results, it’s imperative that outputs from ChatGPT are reviewed and validated by medical professionals. Thus, while ChatGPT could serve as an assistive tool in generating diagnosis, it should not be considered a replacement; rather, radiologists must ensure the accuracy of the diagnoses.

This study assessed the diagnostic accuracy, completeness, and text quality of conclusions produced by ChatGPT. In addition, for neoplastic/cystic diseases, the consistence of ChatGPT’s diagnosis with pathological diagnosis was also evaluated. The results emphasized ChatGPT’s potential in generating diagnoses, particularly in terms of completeness and text quality. Consequently, ChatGPT could potentially be utilized as a supportive tool in future radiology report writing. However, it should be noted that this study was based on a single prompt, and the text evaluation, reliant on a five-point Likert scale, was somewhat subjective.

Since the ChatGPT used in this study is a text-based AI model, it is incapable of direct interpreting radiologic images. Consequently, descriptive radiologic findings (text data) were employed to generate the final diagnoses. Future researches may benefit from integrating image segmentation and image captioning AI models to produce descriptive radiologic findings, which can then serve as the basis for subsequent diagnostic inferences by ChatGPT [25, 26]. Image captioning is the task of describing the visual content of an image in natural language, employing a visual understanding system and a language model capable of generating meaningful and syntactically correct sentences [27]. Furthermore, the recent released ChatGPT4V has allowed for input of images along with text. All these AI models may bring about more changes in radiological report writing.

This study still has several limitations. Firstly, it relied on a restricted dataset that didn’t fully capture the diversity of dental and maxillofacial diseases. The model’s accuracy could fluctuate depending on the complexity, rarity, or specifics of the cases. And only 102 cases were analyzed in this study. Future studies with larger sample sizes are necessary for validation, and these should consider incorporating a more diverse dataset. Secondly, this study used the chief complaint and CBCT radiologic findings as input. To ensure the quality and consistency of the CBCT radiologic findings input, all the CBCT radiologic findings were provided by a radiologist with 10 years of experience and reviewed and modified by a senior radiologist with 15 years of experience. Although ChatGPT produced relatively accurate diagnostic results in this study, it’s important to note that radiologic findings in radiologic reports may vary in real-world conditions due to differences in expertise among radiologists. Such variations could significantly influence the diagnoses generated by ChatGPT. Lastly, this study used only one prompt. As different prompts can significantly impact the outputs [16], further studies using more prompts and compare the outputs of these prompts are needed in future.

## Conclusion

Our study reveals the potential of ChatGPT in generating radiologic diagnoses, demonstrating good diagnosis completeness and text quality. However, achieving diagnostic accuracy, particularly in the context of complex medical issues, remains a challenge. The model’s performance is variable, depending on the complexity of the task, and professional oversight is still crucial due to a certain degree of error rate. Future research based on a more diverse dataset is needed to validate ChatGPT’s effectiveness under real-world conditions.

## Data Availability

The [.xlsx] data and [.docx] used to support the findings of this study were supplied by [Zitong Lin] under license and so cannot be made freely available. Requests for access to these data should be made to [Zitong Lin, E-mail: linzitong_710@163.com].
